# Teledermatology in Low-Resource Settings: The MSF Experience with a Multilingual Tele-Expertise Platform

**DOI:** 10.3389/fpubh.2014.00233

**Published:** 2014-11-14

**Authors:** Sophie Delaigue, Jean-Jacques Morand, David Olson, Richard Wootton, Laurent Bonnardot

**Affiliations:** ^1^Dermatology Department, Hopital Nord, Marseille, France; ^2^Department of Dermatology, Sainte Anne Military Hospital, Toulon, France; ^3^Médecins Sans Frontières, New York, NY, USA; ^4^Norwegian Centre for Integrated Care and Telemedicine, University Hospital of North Norway, Tromsø, Norway; ^5^Faculty of Health Sciences, University of Tromsø, Tromsø, Norway; ^6^Fondation Médecins Sans Frontières, Paris, France; ^7^Department of Medical Ethics and Legal Medicine (EA 4569), Paris Descartes University, Paris, France

**Keywords:** telemedicine, telehealth, dermatology, LMICs, low-resource settings

## Abstract

**Introduction:** In 2010, Médecins Sans Frontières (MSF) launched a tele-expertise system to improve the access to specialized clinical support for its field health workers. Among medical specialties, dermatology is the second most commonly requested type of tele-expertise. The aim of the present study was to review all MSF teledermatology cases in the first 4 years of operation. Our hypothesis was that the review would enable the identification of key areas for improvement in the current MSF teledermatology system.

**Methods:** We carried out a retrospective analysis of all dermatology cases referred by MSF field doctors through the MSF platform from April 2010 until February 2014. We conducted a quantitative and qualitative analysis based on a survey sent to all referrers and specialists involved in these cases.

**Results:** A total of 65 clinical cases were recorded by the system and 26 experts were involved in case management. The median delay in providing the first specialist response was 10.2 h (IQR 3.7–21.1). The median delay in allocating a new case was 0.96 h (IQR 0.26–3.05). The three main countries of case origin were South Sudan (29%), Ethiopia (12%), and Democratic Republic of Congo (10%). The most common topics treated were infectious diseases (46%), inflammatory diseases (25%), and genetic diseases (14%). One-third of users completed the survey. The two main issues raised by specialists and/or referrers were the lack of feedback about patient follow-up and the insufficient quality of clinical details and information supplied by referrers.

**Discussion:** The system clearly delivered a useful service to referrers because the workload rose steadily during the 4-year study period. Nonetheless, user surveys and retrospective analysis suggest that the MSF teledermatology system can be improved by providing guidance on best practice, using pre-filled referral forms, following-up the cases after teleconsultation, and establishing standards for clinical photography.

## Introduction

Telemedicine is broadly defined as any kind of medical activity where distance is involved (1). Tele-expertise, as defined in the French Public Health Code, is one of the five main areas of telemedicine (see Table 1) (2). Telemedicine applications can be divided into two types, according to their mode of information transmission: synchronous (or real time, e.g., videoconferencing) and asynchronous (or store-and-forward, e.g., email).

**Table 1:** **Five areas of telemedicine as defined in the French public health code [2]**.

Area	Comment
Teleconsultation	Consultation at distance between a doctor and a patient
Tele-assistance	Doctor assists another health professional in performing specific procedure
Telemonitoring	Doctor interprets at distance patient data
Medical emergency call center	Triage of calls from the general public, usually by telephone
Tele-expertise	Dialog between treating doctor and a specialist

There is evidence in the literature showing that telemedicine is useful in low-income countries, both for educational and clinical purposes (3). In low-resource settings, there is a chronic shortage of specialists (4), and it has been shown that telemedicine can improve the quality and accessibility of medical care (5) while avoiding costly referrals (6, 7). Telemedicine also has valuable benefits in reducing the isolation of field doctors (8) and facilitating distance education for field health workers who frequently have no other opportunity to access specialized training.

In 2010, Médecins Sans Frontières (MSF) launched a telemedicine project (Box 1) with the aim of improving access to specialized clinical support for its field health workers. The MSF tele-expertise network is based on the Collegium Telemedicus (9) design. It uses a web-based messaging system hosted on a secure server, and store-and-forward methods, which appear to be more appropriate (10) than real time systems in resource-limited settings, because the quality of Internet connection and cost is critical in a humanitarian context (11).

Box 1 **MSF tele-expertise system.**

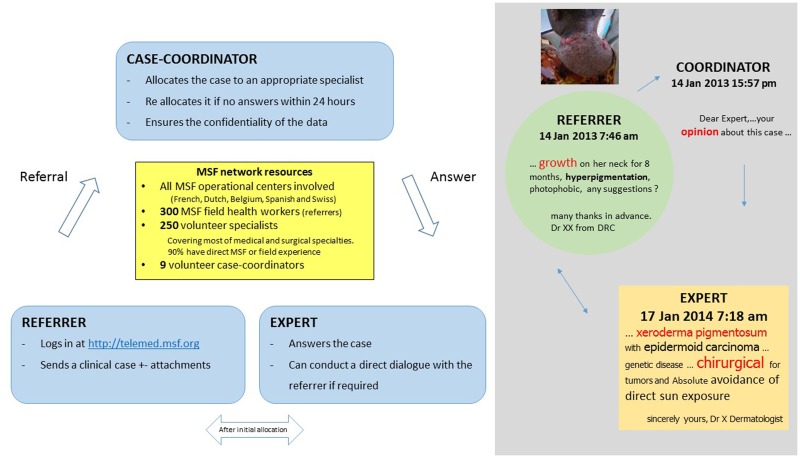

The MSF tele-expertise network has supported a total of 1039 clinical cases, to date, in a wide range of specialties. The three main specialties treated in the MSF telemedicine system are in descending order, radiology, pediatrics, and medical specialties (12). Among the medical specialties, dermatology is the second most common medical specialty after infectious diseases in terms of the number of queries. Thus, dermatology has an important place in the system, which justifies the present analysis.

The aim of the present study was to review all MSF teledermatology cases in the first 4 years of operation. Our hypothesis was that the review would enable the identification of key areas for improvement in the current MSF teledermatology system.

## Materials and Methods

We conducted a retrospective analysis of all dermatological cases referred by MSF field doctors to the MSF telemedicine platform from April 2010 to February 2014.

All cases classified by the IT system as dermatological were retrieved automatically (group 1). To be exhaustive, a manual check of the database was then performed by identifying the most active expert profiles (using the function “user case history”) and all potential cross specialties such as internal medicine, infectious diseases, pediatric, and ear nose throat (ENT) in order to extract all dermatological cases (group 2).

### Workload

We performed a descriptive analysis involving the assessment of the cases submitted. First, we extracted data about system performance, as follows: the number and language of cases, the median delay in providing the first specialist response, and the median delay in allocating a new case. Then, we extracted information on case characteristics, including the countries of origin of the experts and their specialties, the most common topics, the age of the patients, the number of images per case, and the number of follow-up reports and cases of poor quality information reported by specialists.

### Images

We also carried out a qualitative analysis of all images uploaded with the cases submitted. The quality of images was scored as poor, average, or good quality by three of the authors selected because they were clinicians. Images were not scored individually but by case, because this was more clinically meaningful. The assessment was made blind and the results were averaged. The score included a technical analysis of the picture based on focus, anatomical perspective, lighting, and composition (primary lesion and overview picture) (13).

### Survey

In May 2014, we carried out a short-anonymous survey of all users involved in the dermatology cases. The survey design was based on a previous, larger survey, with 50 questions, established after a literature search combined with qualitative data collection. This survey showed that telemedicine was helpful and improved the management of the patient. We wanted to focus some of these questions on dermatological topics. We focused on (i) follow-up because in this large survey it was one of the main lessons learnt and (ii) quality of the referral because the quality of the expertise depends on the quality of the information send (14).

The survey had six questions for the referrers and seven questions for the specialists (closed-ended, opened-ended, scale type, and multiple choice questions). The questions focused on the quality of the referral and follow-up of the patient (Tables 2 and 3). Versions of the survey were made available in French and English. Web-based software (https://www.surveymonkey.com/) was used for collecting the data. Responses were anonymous.

**Table 2:** **(A) Specialist responses (response rate 13/26 = 50%), (B) referrer responses (response rate 9/22 = 41%)**.

**(A)**					
Q1. Was the information (including any images) supplied by the referrer adequate?	Yes		No		Skipped
	8 (62%)		5 (38%)		0
Q2. Was the information about the hospital available on the website (number of doctor, tests)?	Absent	Not sufficient	Sufficient	Easily accessible and complete	1
	2 (15%)	5 (38%)	5 (38%)	0	
Q3. Did you receive any follow-up information about this patient?	Yes		No		0
	1 (7%)		12 (93%)	
Q4. Do you think that feedback about the patient is?	Optional	Desirable	Necessary	Mandatory	0
	1 (7%)	3 (23%)	4 (31%)	5 (39%)	
Q5. Generally speaking how would you rate your satisfaction of the system on scale from 1 to 10?^a^	Average rating				0
	6.37			
Q6. In your opinion, which area(s) of improvement could be fruitful to the MSF teledermatology system?	Establishing formalized guidance for users	Implement a compulsory follow-up process	Conceiving standardized teledermatology pre-filled forms	Request some picture standard	1
	4	8	7	6	
**(B)**					
Q1. Have you ever personally used the system?	Yes		No		Skipped
	6 (67%)		3 (33%)		0
Q2. Have you heard about any follow-up of that (these) case(s)?	Yes		No		0
	5 (56%)		4 (44%)	
Q3. Do you think that feedback about the patient is	Optional	Desirable	Necessary	Mandatory	0
	0	4	4	1	
Q4. Do you think that it is for you?	Impossible	Difficult	Easy	Very easy	1
	1	3	4	0	
Q5. In your opinion when would it be relevant to receive a compulsory follow-up process?	After 1 week	After 2 weeks	After 1 month	After 3 months	1
	5	0	3	0	

*^a^Scale from 0 = not happy at all to 10 = extremely happy with it*.

**Table 3:** **(A) Summary of specialist comments (open-ended questions), (B) summary of referrer comments (open-ended questions)**.

	Number of comments
**(A)**	
Lack of feedback about patient follow-up	3
Lack of information about the case (image, medical history)	3
Against any mandatory system follow-up	1
Annual meeting	1
Proposal to use other technology (e.g., SMS)	1

**(B)**	
Lack of well-adapted answer	1
Lack of epidemiological knowledge of the country of residence	1
Lack of headquarters’ support in using the system	1

### Confidentiality and security

Ethics permission was not required, because patient consent to access the data had been obtained and the work was a retrospective chart review conducted by the organization’s staff in accordance with its research policies (15). Before a new case could be submitted, the referrer had to indicate agreement with the statement “I confirm that informed consent has been obtained from the patient about making an E-referral and its consequences.”

Photography is an important tool for diagnosis in dermatology. To ensure patient privacy, several safeguards are implemented in the telemedicine system. First, the referrer is required to avoid transmitting any identifying data (e.g., the patient’s name). Second, the coordinator ensures that this is respected when he/she allocates the case. Finally, it is recommended that clinical photographs be anonymized by, for example, putting a black bar over the patient’s eyes.

The Collegium Telemedicus system uses secure messaging. Messages are encrypted bidirectionally and are stored on the server; they are only available to the user via a secure SSL connection (10).

## Results

### System performance

**Figure 1:**
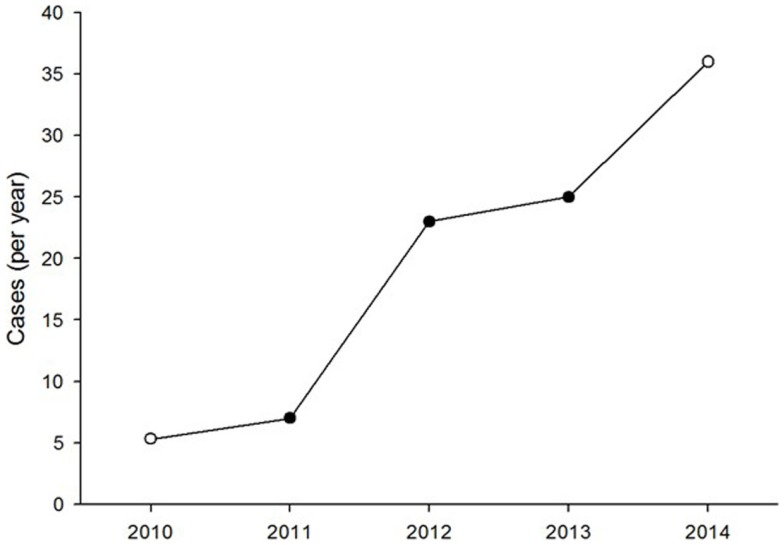
**Number of clinical dermatology cases referred each year**. The open symbols represent values extrapolated from part-year observations.

During the study period, 65 clinical cases from 24 countries were handled by the system. There was a steady increase in the caseload over the 4 year period (Figure 1). Seventy-one percent of the cases were referred in English and 29% in French. No case was submitted in Spanish.

The median delay in providing the first specialist response to the referrer was 10.2 h (IQR 3.7–21.1). The median delay in allocating a new case was 0.96 h (IQR 0.26–3.05). The majority (83%) of the case allocations were done by two case-coordinators.

### Case characteristics

**Figure 2:**
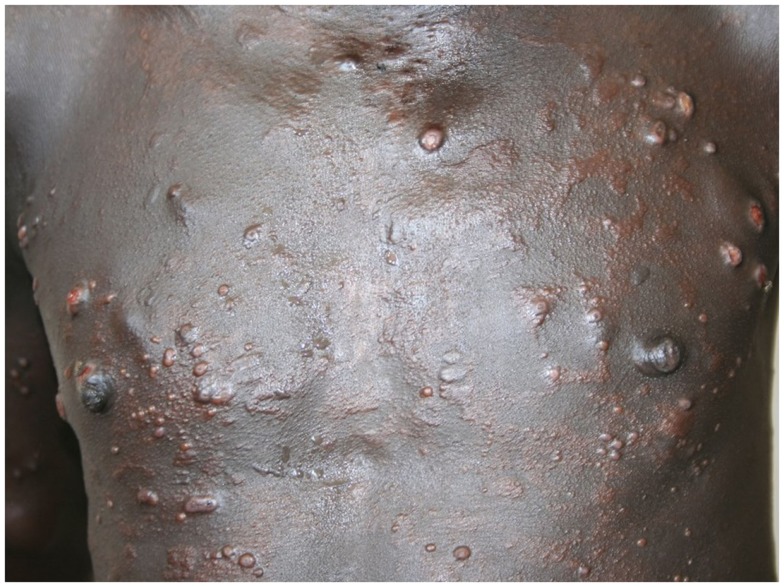
**Confirmed histoid leprosy**.

**Figure 3:**
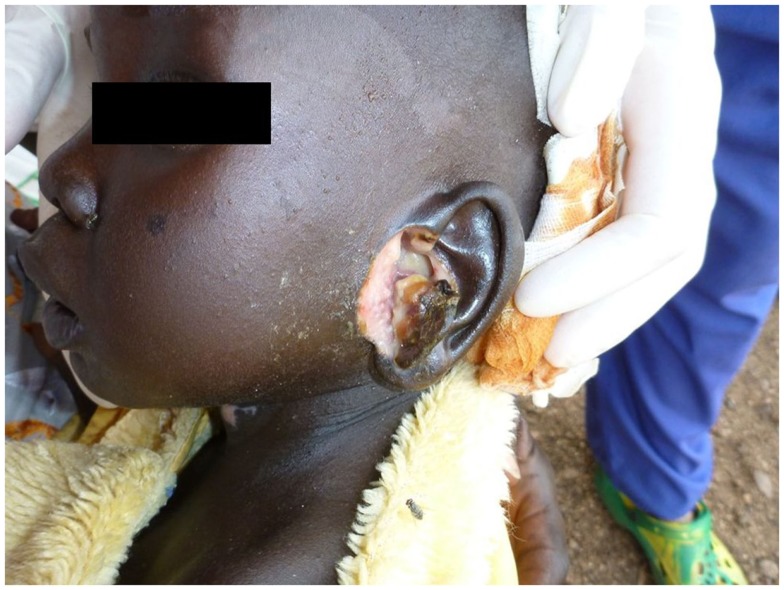
**Suspected mycobacterial infection**.

**Figure 4:**
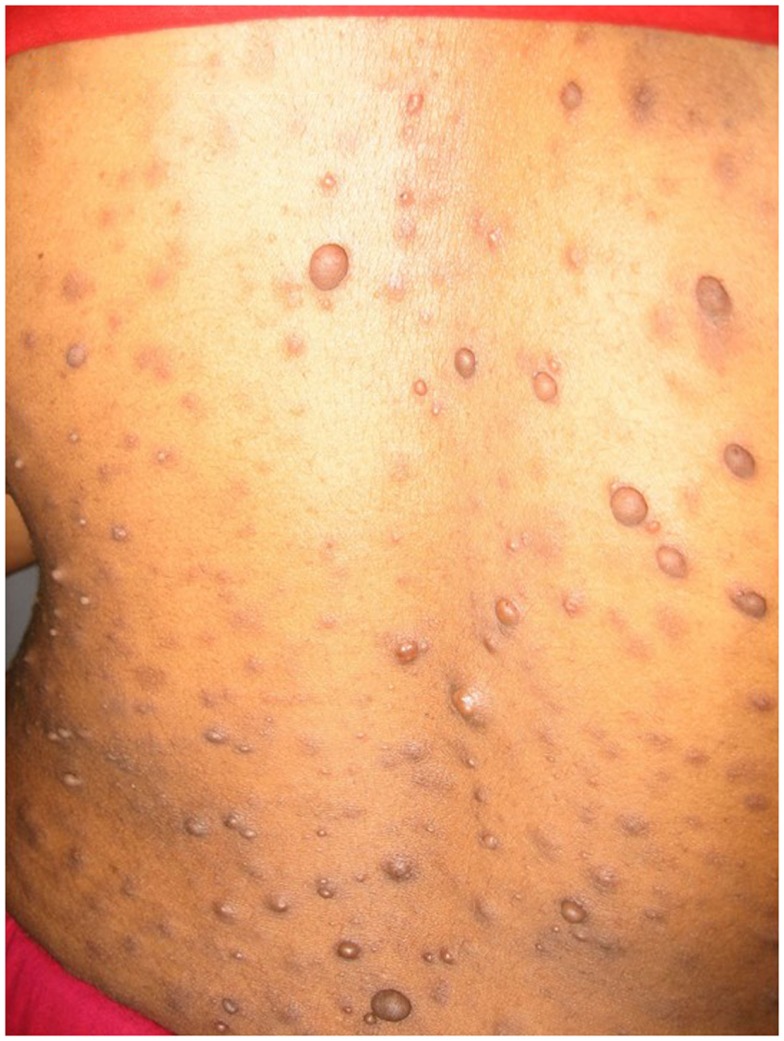
**Neurofibromatosis**.

Most of the cases were focused on diagnosis issues. Among the 65 cases, 43 were tagged as dermatological cases (i.e., group 1) and 22 were cross-specialty cases (i.e., group 2). The three main specialties involved in these cases were infectious diseases (10), pediatrics (8), and internal medicine (4). Examples of these cross-specialty cases are given in Figures 2–4.

**Figure 5:**
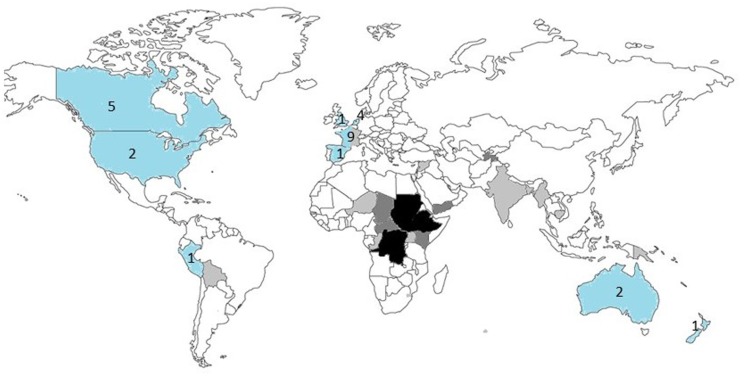
**Countries of origin of the referrers (*n* = 41) and specialists (*n* = 26)**. The countries of origin of the referrers are shaded: light gray = 1 case, dark gray = 2–5 cases, black > 5 cases. The countries of origin of the specialists are shaded in blue, with the number of specialists for each country shown.

The three main countries of case origin were South Sudan (29%), Ethiopia (12%), and Democratic Republic of Congo (10%). Africa (74%) was the main continent of case origin. There were small numbers of cases from Kenya, Yemen, Haiti, Bolivia, India, Cambodia, and Central African Republic (CAR) (Figure 5). The countries of origin of the experts were, in descending order, France (9), Canada (5), the Netherlands (4), USA (2), Australia (2), Peru (1), New Zealand (1), Spain (1), and the UK (1). Experts were specialized in pediatrics (11), dermatology (5), internal medicine (6) plastic surgery (1), general surgery (1), ENT surgery (1), and infectious diseases (1). Countries of origin of both referrers and specialist are shown in Figure 5.

**Figure 6:**
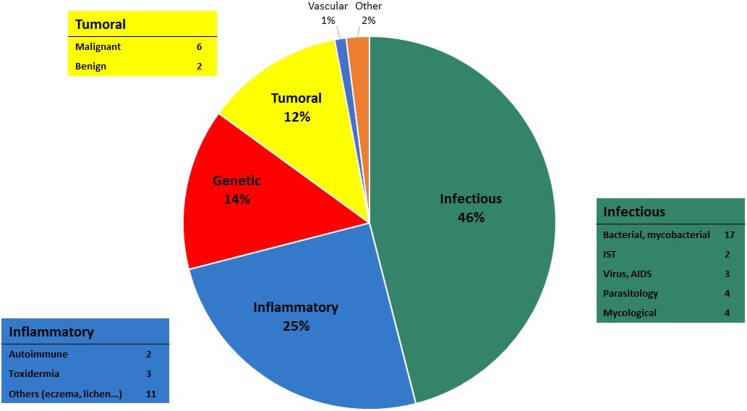
**Most common topics of the cases**.

The most common topics treated were infectious diseases (46%), inflammatory diseases (25%), genetic diseases (14%), and tumor diseases (12%) (Figure 6). Bacterial and mycobacterial infections were the two main sub-topics of infectious diseases. Slightly more than half of the cases (51%) were pediatric (under 18 years old).

A total of 216 images were uploaded with the 65 cases submitted and were reviewed by three specialists. The majority of the images attached were of type JPEG – Joint Photographic Expert Group – (52 cases, 84%), there were 8 cases with compressed files (zip), 2 had copied their images into a Word document and 3 had no attached files. The median number of images per case was 3 (IQR 2, 5). The median file size was 345 kb (IQR 101–1593). Moreover, in 4 cases of the 65, pictures attached were not properly anonymized (patient’s names were mentioned).

The overall quality of the attached pictures were judged by three of the authors as poor quality in 15%, as sufficient for establishing a correct diagnosis in 53% and as good quality in 32%. In addition to that, experts mentioned in 15% of cases in their answers that the pictures sent were of poor quality.

Only 10 (15%) cases had follow-up data. Two reported the death of the patient. The lack of information about patient follow-up was a critical issue and represents a limitation of the analysis.

Fourteen percent of cases were reported as poor quality or not good enough to make a diagnosis.

### Survey results

The survey was sent to 41 referrers and 26 specialists involved in the cases. The survey was completed promptly within 6 days by 22 users (33%: 9 referrers and 13 specialists). Proportionally, more experts completed questionnaires than referrers – 50% (13/26) versus 22% (9/41), respectively. Responses from French and English users were analyzed together.

The principal concern raised by referrers and specialists was the lack of feedback about patient follow-up. Very few experts received any follow-up information about the patients for whom they gave a second opinion, although 56% of the referrers managed to obtain follow-up of referred cases. According to referrers, the optimum period for sending a follow-up report was 1 week.

The second concern raised by specialists was the quality of the clinical details and information supplied by referrers. Summaries of the main referrer and specialist comments made in response to the open-ended questions are shown in Table 3.

## Discussion

The present study reviewed all MSF teledermatology cases in the first 4 years of operation with the aim of identifying areas for improvement. The system clearly delivered a useful service to referrers because the workload rose steadily during the study period. The two main concerns raised by the users were a lack of follow-up information and the quality of the information provided in a referral.

### Limitations of the study

Due to the lack of follow-up and feedback from the field, we were not able to conduct a proper case content analysis in order to assess the overall impact of the system. Although a bigger survey would be better, the aim of the present study was a descriptive analysis. The survey was conducted in order to provide data about improving the system. The chosen topics (feedback and quality of the referrals) were based on existing information. First, the question of feedback was based on another larger survey, which confirmed that lack of feedback is an important source of weakness (12). We wanted to know if this was the case for dermatology. Second, we wanted to assess the quality of the referrals, as it is well established that there is a direct link between the quality of medical records and the quality of healthcare (14).

The high number of experts (26) involved in case management compared to the relatively small number of cases contributed to the heterogeneity of the results and the difficulty in drawing clear conclusions.

Finally, we were not able to compare the telemedicine system to other methods of accessing specialist dermatology advice because there is no structured system within MSF for doing so – only individual practice. There are also very few studies on this topic in low-resource settings with which to compare.

### Useful tool

With more than 15 years’ experience, it is now clearly established that this kind of system is reliable, efficient, and easy to use for doctors working in developing countries (16). Like other long-running telemedicine networks delivering humanitarian medical services such as the Africa teledermatology project (17), the MSF telemedicine system confirms that teledermatology is an important area of use and development. There is nothing surprising in this observation as this specialty is mainly based on visual diagnosis and dermatology conditions are often manifestations of underlying illnesses, such as infectious disease, which can have its own specialist input, making dermatology a particularly good fit for tele-expertise.

**Figure 7:**
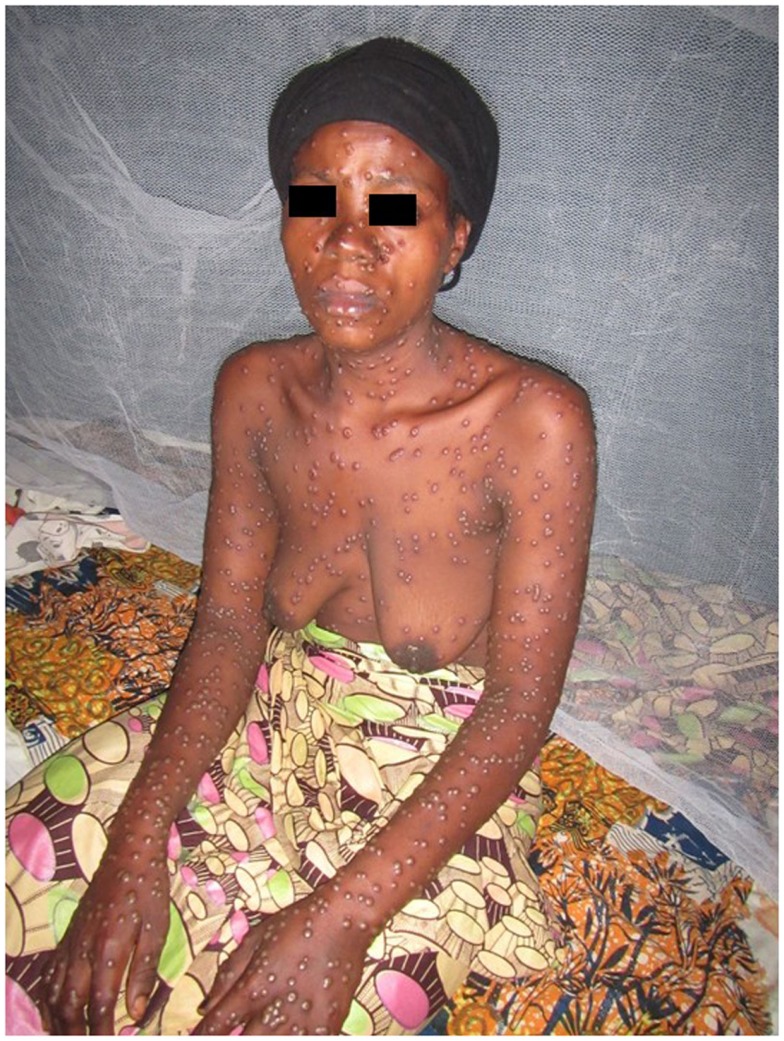
**Suspected pox virus infection**.

The increase in the number of referrals over the past 4 years is a sign of vitality and confirms the growing need for such a system. It therefore seems crucial to enhance the MSF telemedicine system to make sure it can absorb this growth while remaining efficient. As there is no other option for obtaining access to a specialist consultant in most of these low resource settings, this kind of system represents a pragmatic and efficient answer to the chronic shortage of specialists. Furthermore, it is worth noting that with a mean delay of 10 h to the first specialist answer, many industrialized countries would be envious of this level of response. This remarkable reactivity from our experts who all work full time in other settings is undoubtedly linked to their motivation in providing assistance to isolated doctors with the shortest delay. But, it is also clear that by dealing with cases out of their usual practice environment, our experts find a personal interest in handling some of the rare and interesting cases referred with professional value both clinical and academic (see for example, Figure 7).

### Weaknesses and recommendations

Despite its positive impact, the present study identified various weaknesses to be addressed to ensure the long-term sustainability of the telemedicine system. We therefore make three main recommendations.

**Figure 8:**
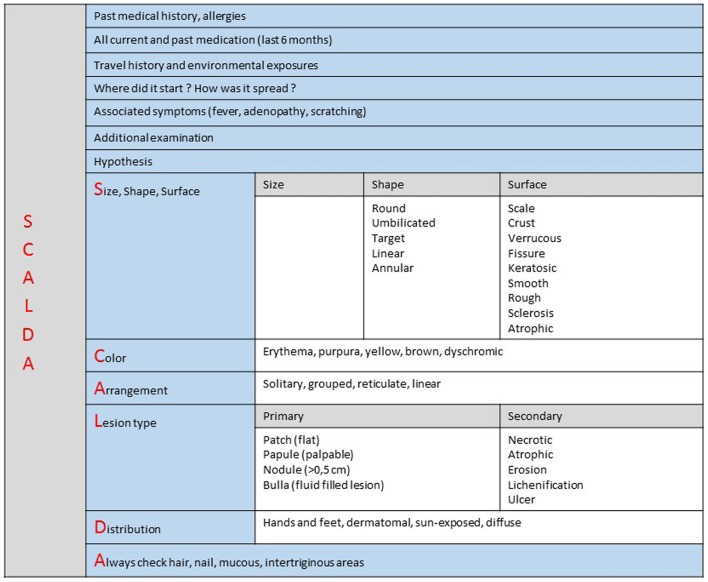
**Dermatology history form**.

**Figure 9:**
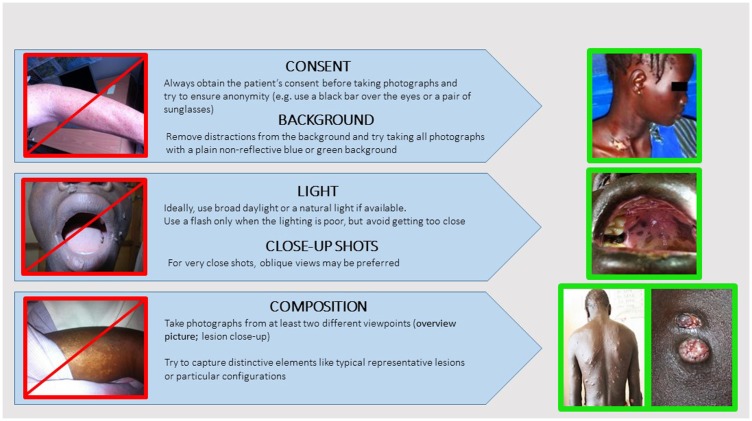
**Dermatology photography recommendations**.

1. *Provide more information about system use*. Looking at the number and heterogeneity of origin of cases – 19 cases from South Sudan while only single cases from many other countries – it is clear that the system has not yet reached its full potential and that widespread field implementation has not yet occurred. Moreover, MSF provides medical aid in some 70 countries around the world, while we recorded teledermatology cases from only 24 countries (18). Strong political commitment and a defined communication strategy from MSF headquarters are necessary to drive the process of implementing a new tool in the field. As stated by the WHO (19) in 2005 concerning e-health in general, establishing formalized guidance for MSF e-dermatology users could reinforce and better structure the system, as well as increase its visibility. Clear information about how and when to use the system (i.e., when facing a difficult clinical case rather than making a general query about guidelines) must be given to users. There should also be a regular update of information about field projects as well as global information about the system (e.g., one suggestion made by users was to hold an annual MSF telemedicine meeting).
2. *Improve the quality of referrals*. The quality of referrals can be improved by standardizing the clinical examination and by establishing standards for photography:
  - *Standardizing teledermatology clinical examination*. Fourteen percent of cases were reported by the expert as poor in quality or not good enough to make a diagnosis. If dermatological diagnosis is mainly clinical – based on visual inspection with no sophisticated investigations required – it is, however, not sufficient to make an accurate diagnosis. Our analysis confirms that non-specialist practitioners do not master dermatological language or basic knowledge that would allow them to send a complete and accurate medical report. A standardized teledermatology structured form could then both facilitate communication and improve diagnosis performed with the MSF teledermatology system (20) (Figure 8). This standardized form has been made available for all new dermatology cases since September 2014.
  - *Establish picture standards*. Fifteen percent of the attached pictures were considered by experts as of poor quality. Picture quality is crucial to reliable teledermatology diagnosis (21). Many studies have shown that when teledermatology relies on pictures of good quality it can deliver the same diagnosis that proper physical examination does in most cases (22). As with a face to face examination, pictures should represent the whole patient before providing close-ups of the primary lesion (23). As a standard, referrers should attach at least two pictures to all dermatology cases referred (one close-up, the other one an overview). Some classic general recommendations to standardize attached pictures are given in Figure 9.
3. *Provide more follow-up information*. One of the main concerns raised by experts in the survey was the lack of patient follow-up information. Only 10 cases (15%) had feedback data recorded. Most of the users agreed that follow-up was necessary (44% referrer majority response) or even mandatory (39% specialist majority response). This is necessary to keep the experts motivated and also to improve the quality of their answers and to allow them to learn from the referrer’s feedback. By systematically recording patient outcomes, we can assess the real benefit, exploit statistics, and conduct proper scientific evaluation. Without feedback, the principle of requesting specialist advice is weakened, because no quality control is feasible. For facilitating the process, follow-up reports have been set up and are sent out automatically to the referrer.

## Conclusion

The present review shows that teledermatology is a growing part of a unique multilingual tele-expertise system supporting health professionals in the management of difficult clinical cases in the field. As shown in a previous larger survey (12), the majority of referrers (79%) reported that the advice received via the system improved their management of the patient. Nonetheless, user surveys and retrospective analysis suggest that the MSF teledermatology system can be improved. These improvements include providing information about system use, improving the quality of referrals and providing more follow-up information after teleconsultation. A future prospective evaluation could assess the impact of these recommendations.

## Conflict of Interest Statement

The authors declare that the research was conducted in the absence of any commercial or financial relationships that could be construed as a potential conflict of interest.
